# Metabolic perturbation reduces antibiotic tolerance in Mycobacterium tuberculosis

**DOI:** 10.1099/mic.0.001697

**Published:** 2026-04-22

**Authors:** Chen-Yi Cheung, Imogen Samuels, Hannah R. Klaus, Gregory M. Cook, Matthew B. McNeil

**Affiliations:** 1 Department of Microbiology and Immunology, University of Otago, Dunedin, New Zealand; 2 School of Biomedical Sciences, Faculty of Health, Queensland University of Technology, Brisbane, Queensland, Australia

**Keywords:** antibiotic, mycobacteria, tolerance

## Abstract

*Mycobacterium tuberculosis* is tolerant to many antibiotics, leading to impaired antibiotic killing. Using CRISPR interference (CRISPRi) transcriptional knockdowns, we generated a panel of metabolically compromised strains to identify tolerance pathways for pursuing in therapeutic development. Disrupting the regulation of intracellular iron storage, amino acid biosynthesis and redox defence mechanisms potentiated the lethality of multiple drugs and translated to infected THP-1 macrophages. This work reinforces the role of metabolism as a major contributor to drug tolerance in *M. tuberculosis.*

## Introduction

*Mycobacterium tuberculosis*, the causative agent of tuberculosis, remains an infectious disease of global significance. A major barrier to reducing therapeutic treatment times is overcoming the intrinsic drug tolerance of *M. tuberculosis* that impairs the bactericidal effect of clinically available antibiotics [1,8]. Chemical inhibitors capable of removing tolerance, ideally to multiple antibiotics, have significant potential as antibiotic adjuvants by offering a potential route to drastically reducing treatment times. Several studies have demonstrated that the inhibition of metabolic or bioenergetic pathways can inactivate tolerance and sensitize *M. tuberculosis* to antibacterial killing [9,11]. This includes the inhibition of the respiratory oxidase cytochrome *bd* oxidase, which allows for the normally bacteriostatic phenotype of cytochrome *bc_1_aa_3_* inhibitors to be bactericidal against *M. tuberculosis* [8,11, 12]. The prioritization of targets to pursue as tolerance inhibitors has been hindered by the need to construct gene deletions, in some cases of essential genes, to validate loss-of-tolerance phenotypes. Here, we sought to address this by using a CRISPR interference (CRISPRi) transcriptional knockdown strategy to generate an array of genetic depletion strains to allow the head-to-head comparisons of potential anti-tolerance pathways. As prior results from both mycobacteria and other bacterial pathogens have implicated metabolic pathways in antibiotic tolerance [2,3, 5,8, 13], we focused on genes involved in (i) specific metabolic pathways, (ii) central cellular processes expected to produce global metabolic disruption and (iii) redox stress response mechanisms.

## Methods

### CRISPRi plasmid construction

To generate a panel of metabolically compromised strains, we constructed CRISPRi plasmids, each with a unique guide RNA (gRNA) targeting a gene involved in a metabolic or biosynthetic pathway, a central cellular function or a redox stress response (Table S1, available in the online Supplementary Material). Plasmids were constructed following previously established Golden Gate cloning protocols [18,19]. Plasmids were transformed into *M. tuberculosis* strain mc26206 using previously established protocols [20].

### CRISPRi phenotypic screen for antibiotic tolerance

We used CRISPRi to pre-deplete strains of specific metabolic targets. This was achieved by inoculating *M. tuberculosis* mc26206 with relevant CRISPRi plasmids into T25 tissue culture flasks that contained 10 ml of 7H9 broth supplemented with OADC (0.005% oleic acid, 0.5%, 0.2% dextrose, 0.085% catalase), 0.05% tyloxapol (Sigma), 25 µg ml^−1^ pantothenic acid, 50 µg ml^−1^ leucine, 25 µg ml^−1^ kanamycin (KAN) and 300 ng ml^−1^ anhydrous-tetracycline (ATc), at a starting OD at 600 nm (OD_600_) of 0.005. Strains were grown for 5 days to pre-deplete targets. Pantothenic acid and leucine were added as all experiments in this study used the avirulent auxotrophic strain *M. tuberculosis* mc^2^6206. After 5 days’ incubation, we separated strains into (a) strains that, following CRISPRi induction, did not severely impair bacterial growth and were diluted 1/10 prior to inoculation into antibiotic assay plates and (b) strains that, following induction of gRNA expression, impaired growth to an OD_600_ of <0.2 and were not diluted prior to inoculation into antibiotic assay plates. Seventy-five microlitres of the relevant culture dilution was added to a 96-well flat-bottomed microtiter plate containing compound and ATc at 300 ng ml^−1^ to achieve a starting OD_600_ of 0.005 in a final volume of 150 µl. Assay plates were prepared as described below. Plates were incubated at 37 °C for 10 days without shaking. Viability was determined on days 0 and 10. Viability at day 0 was determined using a four-point ten-fold dilution of the relevant diluted culture, with 5 µl of each dilution spotted onto 7H11 agar plates. At day 10, the culture was removed from appropriate wells and transferred to a new 96-well plate for dilution. A four-point ten-fold dilution gradient was performed, and 5 µl of each dilution was spotted onto 7H11 agar plates. Plates were incubated at 37 °C for 4–5 weeks, and colonies were counted.

### Compound susceptibility assays and plate setup

Briefly, inner wells (rows B–G, columns 3–11) of a 96-well flat-bottomed microtiter plate (Thermo Fisher Scientific) were filled with 75 µl of 7H9 media. Outer wells were filled with 150 µl of 7H9 media as media-only controls, and 113 µl of 7H9 media containing the compound of interest at the required starting concentration was added to column 2 of rows B–G. The compound was diluted threefold, by transferring 37.5 µl between wells, down to column 10. Column 11 was kept as solvent-only control. For susceptibility assays, strains were diluted to an OD_600_ of 0.01. Seventy-five microlitres of diluted culture was added to inner wells of the 96-well flat-bottomed microtiter plate containing the compound to achieve a starting OD_600_ of 0.005 in a final volume of 150 µl. Plates were incubated at 37 °C for 10 days without shaking. After 10 days, plates were covered with plate seals, shaken for 1 min, and the OD_600_ was determined using a Varioskan Flash microplate reader (Thermo Fisher Scientific). OD_600_ readings from duplicate plates were corrected for background, and values relative to the growth of the no-ATc control were analysed using a nonlinear fitting of the data to a Gompertz equation. For viability assays, CRISPRi pre-depletion strains were prepared as described above, with viability determined on days 0 and 10.

### Macrophage infection assays

THP-1 (a human monocytic macrophage-like cell line) macrophage infection studies were performed using previously described protocols [21]. Briefly, the human monocytic cell line THP-1 (ATCC Cat# TIB-202) was cultured in standard RPMI 1640 macrophage medium supplemented with 10% inactivated FBS and 1 mM sodium pyruvate at 37 °C with 5% CO_2_. THP-1 monocytes (5×10^5^ cells per well) were differentiated overnight using 100 ng ml^−1^ phorbol myristate acetate and seeded in a 24-well plate. Differentiated macrophages were infected with a mid-logarithmic-phase culture of *M. tuberculosis* with a CRISPRi plasmid (OD 0.4–0.8) at a multiplicity of infection of 10 : 1 (10 bacteria/1 cell). Infection was allowed to proceed for 1 h. Cells were then washed three times with pre-warmed complete RPMI to remove extracellular bacilli. RPMI media containing supplements (pantothenic acid 25 µg ml^−1^ and leucine 50 µg ml^−1^), 0.1% BSA, 300 ng ml^−1^ ATc and antibiotics at defined concentrations were added to the infected cells and incubated at 37 °C with 5% CO_2_. After 3 days, infected cells were lysed in distilled water containing 0.1% tyloxapol for 5 min at room temperature to determine the number of c.f.u. ml^−1^ on 7H11 agar supplemented with KAN. The percentage of cell viability was determined by normalizing c.f.u. ml^−1^ counts at day 3 following compound treatment relative to the inoculum, as determined on day 0.

## Results and discussion

**Fig. 1.**
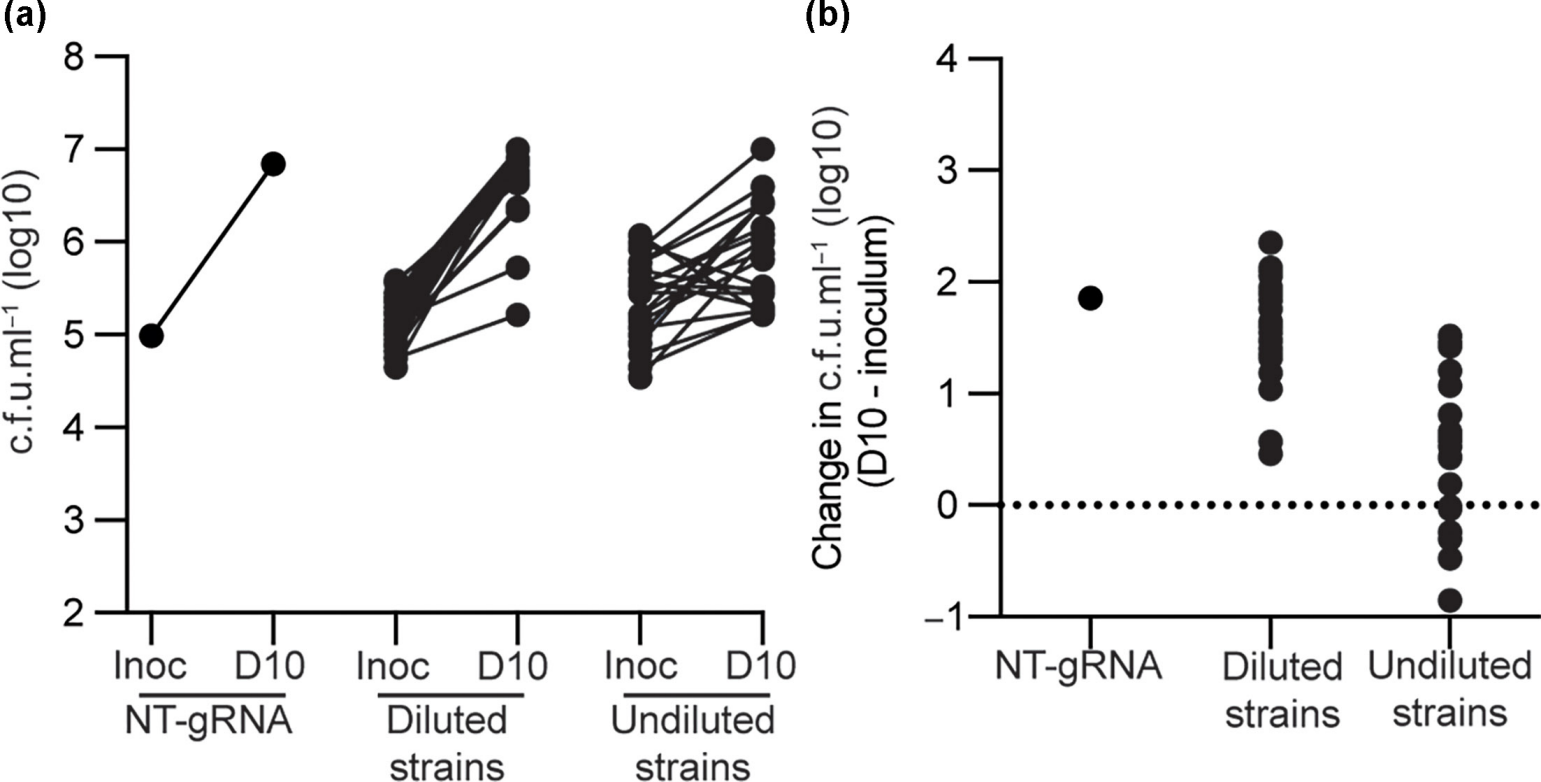
A panel of metabolically compromised strains of *M. tuberculosis*. Using CRISPRi, we pre-depleted genes in *M. tuberculosis* for a period of 5 days before inoculation into 96-well assay plates that contained 75 µl of 7H9 supplemented media containing 300 ng ml^−1^ ATc. gRNAs targeting non-essential pathways were diluted 1/10 prior to inoculation, whilst those targeting essential genes or previously shown to be growth inhibiting were not diluted. In both cases, 75 µl of culture was inoculated into assay plates and incubated for 10 days to determine effects of transcriptional knockdown on viability. Bacterial viability was determined by plating for c.f.u.s and expressed as (**a**) log_10_ c.f.u. ml^−1^ and (**b**) change in c.f.u. ml^−1^ relative to the starting inoculum. The data used to generate 1b are provided in Table S1. NT-gRNA, non-targeting gRNA, inoc: colony forming units at innoculum, D10: colony forming units at day 10.

To generate an array of metabolically compromised strains, we targeted 47 genes involved in (i) specific metabolic pathways, (ii) central cellular processes expected to produce global metabolic disruption and (iii) redox stress response mechanisms. Whilst not an exhaustive list, the targets selected are important contributors to each of these pathways. Consistent with prior approaches, for gRNAs that had known bactericidal phenotypes (i.e. *pyrG*, *rpoB* and *groES*), we used suboptimal gRNA sequences that had reduced killing phenotypes to enable the quantification of synergistic interactions in chemical-genetic screens [18,22]. For many of the gRNA used in this study, prior transcriptional quantification had confirmed that they are on-target and repress target genes [18,20,22, 23]. CRISPRi also provides advantages over conventional genetic deletions, as the construction of transcriptional knockdown mutants is less resource-intense, and genes with essential or strong fitness phenotypes can be included. Furthermore, the length of time required to generate genetic deletions provides *M. tuberculosis* with a potential opportunity for metabolic adaptation to created mutations, potentially obscuring the contribution of target pathways to antibiotic tolerance. Conversely, CRISPRi transcriptional knockdowns used to pre-deplete *M. tuberculosis* of a specific target prior to antibiotic challenge are less likely to provide time for these metabolic adaptations to occur. CRISPRi can also have polar effects on downstream co-transcribed genes [19]. Whilst CRISPRi can be subject to potential off-target effects, prior genome-scale CRISPRi studies have demonstrated that the *Streptococcus thermophilus* sth1 type Cas9 used in mycobacterial CRISPRi had no detectable off-target effect, possibly due to a longer Protospacer adjacent motif (PAM) sequence compared with the conventional *Streptococcus pyogenes* Cas9 [24]. Following initial CRISPRi to pre-deplete the target of interest, we separated strains into (a) strains that, following CRISPRi induction, did not severely impair bacterial growth and were diluted 1/10 prior to inoculation into antibiotic assay plates and (b) strains that, following induction of gRNA expression, impaired growth to an OD_600_ of <0.2 and were not diluted prior to inoculation into antibiotic assay plates. All strains were inoculated into assay plates at a starting inoculum of ∼10^5^ c.f.u. ml^−1^ (Fig. 1a). Over a 10-day incubation period in 96-well plates with ATc exposure, viable colonies increased for the negative control that expressed a scrambled non-targeting gRNA and most diluted strains, consistent with them targeting largely non-essential pathways (Fig. 1a, b). Undiluted strains had more variable effects on viability, with many strains showing no change or a small reduction in viable counts (Fig. 1a, b). This is consistent with these gRNAs targeting largely essential genes or genes with a strong fitness phenotype. The separation of strains into those with and without a growth phenotype following initial pre-depletion is unlikely to be a reflection of differences in transcriptional knockdown, as prior work from us and others has demonstrated that there is no correlation between the level of transcription knockdown and phenotype [18,24, 25]. Phenotype is instead a reflection of differences in genetic vulnerability and phenotypic buffering [18,24, 25]. In conclusion, CRISPRi can be used to generate a panel of metabolically compromised strains of *M. tuberculosis*.

**Fig. 2.**
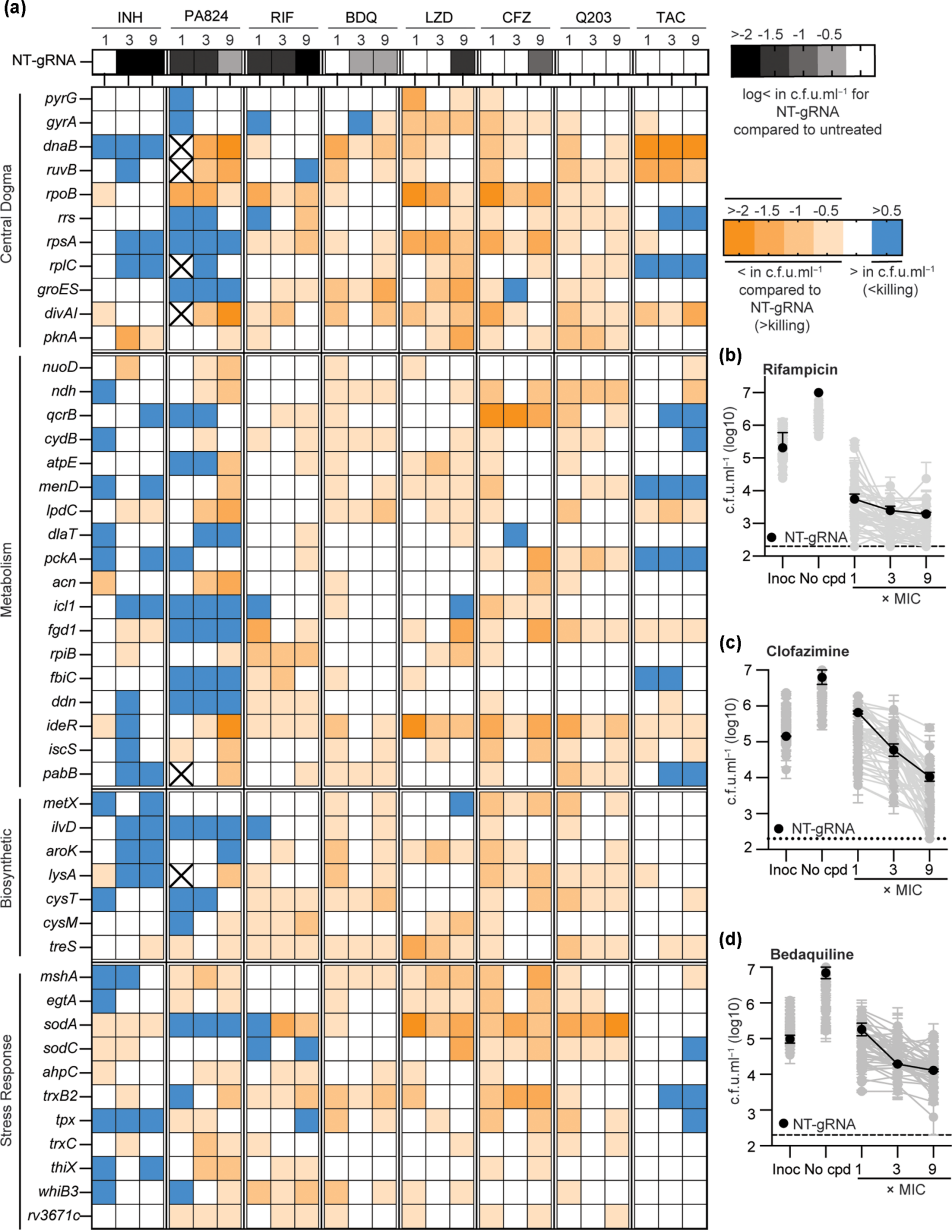
Metabolically compromised strains of *M. tuberculosis* alter sensitivity to antibiotic killing. (**a**) Killing of metabolically compromised strains after 5 days of CRISPRi-mediated pre-depletion of the stated target. The level of killing was determined after 10 days by plating for viable colonies. The killing of the NT-gRNA is noted along a grey scale heatmap. Altered killing of depleted strains is noted as blue for reduced killing and orange for increased killing relative to the non-targeting control. All data represent the combined average of biological duplicates from at least two replicate experiments. (**b–d**) Examples of the c.f.u. ml^−1^ outputs from experiments with RIF, clofazimine and bedaquiline used to generate the data are shown in panel (**a**). The NT-gRNA control is highlighted as black circles, whilst all other strains are represented as grey circles. Inoc represents the seeding c.f.u. ml^−1^ to start each experiment following target pre-depletion. No cpd represents how pre-depleted strains grow in assay plates in the absence of antibiotic over a 10-day period. CFZ, clofazimine; INH, isoniazid; LZD, linezolid; NT-gRNA, non-targeting gRNA; PA824, pretomanid; TAC, thioacetazone; Q203, Telacebec.

Using our pre-depletion strategy, metabolically compromised strains were inoculated into assay plates and exposed to increasing drug concentrations for 10 days. To assess the influence of metabolic perturbation on antibiotic tolerance, cell viability was determined at compound concentrations that corresponded to 1×, 3× and 9× MIC of the non-targeting negative control by plating for viable colonies using established methods (Fig. 2a) [20]. Of the eight compounds tested, isoniazid and rifampicin (RIF) had the strongest bactericidal activity against the non-targeting control, with a >2 log reduction in c.f.u. ml^−1^ at 9× the MIC (Fig. 2a). PA824 (pretomanid) was bactericidal, yet had reduced bactericidal activity at 9× MIC, possibly due to the selection of resistance clones (Fig. 2a). Bedaquiline, linezolid and clofazimine were bactericidal, although to a lesser extent than isoniazid and RIF in terms of both magnitude and the concentrations at which they were bactericidal (Fig. 2a). Q203 (Telacebec) and thioacetazone were bacteriostatic (Fig. 2a). Consistent with prior reports, bioenergetic targets *ndh, qcrB* and *menD* perturbed the killing of isoniazid [26]. The inhibition of amino acid biosynthesis potentiated antibiotic killing, although there was variability in the antibiotics and the level of potentiation (Fig. 2a). For example, only the inhibition of cysteine biosynthetic genes *cysT* and *cysM* potentiated RIF killing (Fig. 2a). Conversely, the inhibition of diverse biosynthetic pathways, including cysteine biosynthetic genes, potentiated the killing of both bedaquiline and clofazimine (Fig. 2a). Increases in reactive oxygen species have been proposed as being a shared mechanism of antibiotic killing [27,29]. Consistent with this, the depletion of pathways that mitigate redox stress increased the lethality of multiple antibiotics, with there being distinct relationships between mitigation pathways. For example, the depletion of thioredoxin biosynthesis (*trxB2*) potentiated RIF, bedaquiline and clofazimine; the depletion of superoxide dismutase (*sodA*) potentiated linezolid, clofazimine and Q203; whilst the depletion of mycothiol potentiated PA824, bedaquiline, linezolid and clofazimine (Fig. 2a). In some instances, there were also divergent effects on killing by a single gRNA, with reduced killing at low concentrations and increased killing at high concentrations (e.g. *sodA* vs RIF) (Fig. 2a). Whilst antibiotic killing is a multifaceted process, these results highlight how disabling redox stress mitigation can potentiate antibiotic killing. In conclusion, perturbing metabolic pathways with transcriptional inhibition allows for the identification of pathways that alter antibiotic killing.

**Fig. 3.**
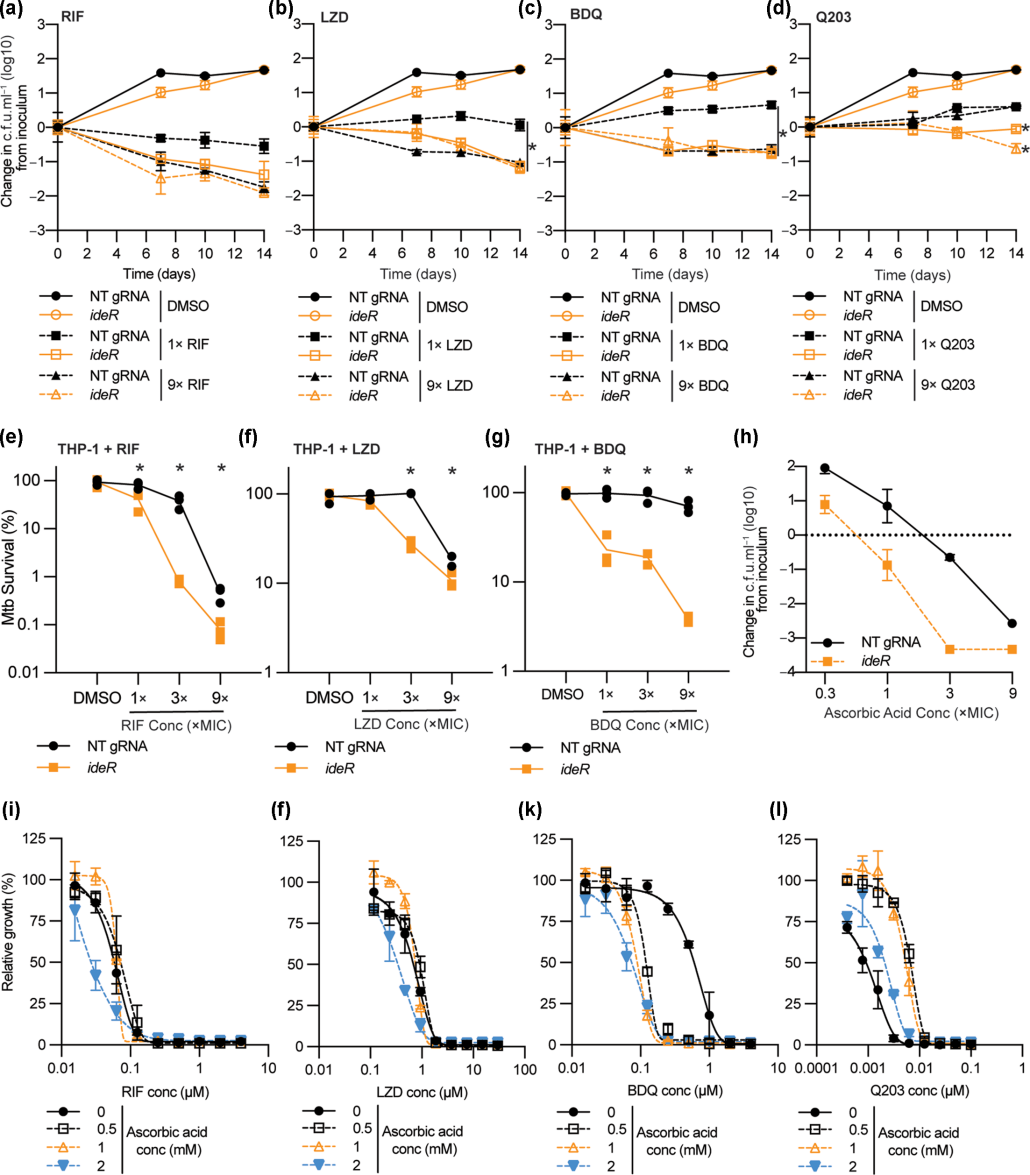
Depletion of *ideR* potentiates killing by a broad range of functionally diverse antibiotics. (**a–d**) CRISPRi was used to pre-deplete *ideR* for 5 days, with the culture then inoculated into 10 ml of 7H9-OADC with compounds at the stated concentrations. The MICs for the compounds are as follows: RIF (0.4 µM), LZD (4 µM), BDQ (0.3 µM) and Q203 (0.02 µM). Bacterial killing was detected by plating for viable colonies at the stated time points and expressed as a reduction in c.f.u. ml^−1^ relative to the inoculum. For (a–d), a t-test was performed on day 14 data comparing the final viability for the *ideR* knockdown relative to the non-targeting control exposed to the same concentration of antibiotic. *P-value <0.05. (**e–g**) *M. tuberculosis* strains pre-depleted of *ideR* were used to infect THP-1 macrophage-like cells using established protocols [21]. Once extracellular cells were removed, infected THP-1 cells were exposed to compounds at the stated concentrations for a period of 3 days. ATc was maintained at 300 ng ml^−1^ for the 3-day period. Bacterial killing was determined by plating for viable colonies and expressed as survival relative to a DMSO-only control. For (E–G), a t-test was performed at each concentration comparing the viability for the *ideR* knockdown relative to the non-targeting control exposed to the same concentration of antibiotic. *P-value <0.05. (**h**) Survival of *M. tuberculosis* pre-depleted of *ideR* when exposed to increasing concentrations of ascorbic acid. Viability was determined after 10 days’ incubation by plating for viable colonies. (**i–l**) Compound synergy experiments. The stated compounds were diluted in a twofold dilution in 96-well plates in media containing no ascorbic acid, or ascorbic acid at 0.5, 1 or 2 mM. Assay plates were prepared as previously described [19,22]. Optical density in each well was determined after 10 days’ incubation, with dose–response curves fitted using the Gompertz equation as previously described [18,34]. Data are expressed relative to a no ascorbic acid control. In all experiments, *M. tuberculosis* strain mc26206 was used. All experiments are the average of at least biological duplicates from at least two replicate experiments. LZD, linezolid; conc, concentration; Q203, Telacebec.

Our initial pre-depletion screen demonstrated that *ideR* potentiated the killing of seven out of the eight tested antibiotics (Fig. 2). To validate these interactions, we performed time-kill assays, in which again *ideR* was pre-depleted for 5 days prior to exposure to 1× or 9× MIC of RIF, linezolid, bedaquiline or Q203, with colony viability being monitored over a period of 14 days. When used at 1× MIC, pre-depletion of *ideR* enhanced bacterial killing of linezolid and bedaquiline by >1 log_10_ c.f.u. ml^−1^ by day 14, whilst RIF killing was enhanced by >0.5 log_10_ c.f.u. ml^−1^ (Fig. 3a–c). For the same three antibiotics, there was no improvement in bacterial killing when used at 9× MIC (Fig. 3a–c). Pre-depletion of *ideR* enhanced the bacteriostatic properties of Q203 at 1× MIC and reduced bacterial viability by ∼0.5 log_10_ c.f.u. ml^−1^ by day 14 compared with the starting inoculum (Fig. 3d). To determine whether these results translated to intracellular infection models, we infected THP-1-like macrophages with *M. tuberculosis* strains pre-depleted for *ideR* using established protocols [21]. Following the removal of extracellular bacteria, antibiotics were added for 3 days, at which point viable colonies were determined. ATc was maintained throughout the 3-day antibiotic exposure. Compared to a negative control, the pre-depletion of *ideR* improved the intracellular killing of RIF, linezolid and bedaquiline across multiple concentrations above the MIC (Fig. 3e–g). Intracellular iron plays a primary role in oxidative stress due to the generation of hydroxyl radicals from the Fenton reaction [30]. *IdeR* is a transcription factor that regulates the expression of many genes involved in iron metabolism and intracellular storage of iron. Consequently, depletion of *ideR* would be expected to have a multitude of transcriptional impacts on a large gene set, including the regulation of proteins required for intracellular iron storage. Furthermore, the depletion of *ideR* increases intracellular iron content and sensitizes *M. tuberculosis* to oxidative stress [31,32]. Vitamin C (i.e. ascorbic acid) has bactericidal activity against *M. tuberculosis*, which both drives and is dependent on high iron levels [30]. Consistent with this, the depletion of *ideR* increased the sensitivity of *M. tuberculosis* to killing by ascorbic acid (Fig. 3h). Because of the overlapping phenotypes between *ideR* and ascorbic acid, we hypothesized that ascorbic acid would have synergistic interactions with antibiotics that had increased efficacy against an *ideR* depletion strain. Consistent with this hypothesis, sub-therapeutic concentrations of ascorbic acid increased the activity of bedaquiline by eightfold (Fig. 3k). This ascorbic acid potentiation of bedaquiline is consistent with recent reports of a metabolically mediated mechanism of synergy [33]. The activity of RIF and linezolid was marginally improved, as evident through a change in the slope of the dose–response curve, although there was no reduction in MIC (Fig. 3i, j). Conversely, the activity of Q203 was antagonized by the addition of ascorbic acid (Fig. 3l). Ascorbic acid has been shown to have multiple effects on mycobacterial physiology, including alterations in NADH:NAD^+^ ratios and enhanced respiration rates [30,33]. Furthermore, synergy between ascorbic acid and bedaquiline is dependent on the availability of iron, as the strength of synergy is reduced in the presence of iron chelators [33]. Synergy with bedaquiline (BDQ) and antagonism with Q203, both of which are bioenergetic inhibitors, likely reflect overlapping (for BDQ) or contrasting (for Q203) effects on mycobacterial physiology. In conclusion, the *ideR*-mediated potentiation of antibiotic killing translates to intracellular conditions and can, to an extent, be exploited by ascorbic acid. These results also expand upon prior observations that the loss of intracellular iron storage in a *M. tuberculosis* ferritin mutant (i.e. Δ*brfB*) leads to a loss of tolerance to killing by multiple antibiotics, including aminoglycosides, fluoroquinolones and isoniazid [32].

Here, we have described the application of CRISPRi to construct a panel of metabolically compromised strains of *M. tuberculosis* to identify high-value targets for unlocking antibiotic tolerance. These results demonstrate that sensitization relationships were highly variable, with some pathways only potentiating a single antibiotic, whilst others had either a large or marginal potentiation against multiple antibiotics. Importantly, these results translated well to intracellular infection models. The combined outputs of this study provide a useful resource for the prioritization of metabolic pathways to target in drug discovery campaigns and the design of antibiotic combinations focused on improving antibiotic killing.

## Supplementary material

10.1099/mic.0.001697 Uncited Table S1.

## Abbreviations

ATc: anhydrous-tetracycline
BDQ: bedaquiline
CRISPRi: CRISPR interference
KAN: kanamycin
OD_600_: OD at 600 nm
RIF: rifampicin
